# Remarks on the Prolactin Hypothesis of Peripartum Cardiomyopathy

**DOI:** 10.3389/fendo.2017.00077

**Published:** 2017-04-11

**Authors:** Jakob Triebel, Carmen Clapp, Gonzalo Martínez de la Escalera, Thomas Bertsch

**Affiliations:** ^1^Institute for Clinical Chemistry, Laboratory Medicine and Transfusion Medicine, Nuremberg General Hospital, Paracelsus Medical University, Nuremberg, Germany; ^2^Instituto de Neurobiología, Universidad Nacional Autónoma de México (UNAM), Querétaro, México

**Keywords:** prolactin, vasoinhibins, peripartum cardiomyopathy, prolactin/vasoinhibin axis, bromocriptine, 16 kDa PRL

A seminal study in 2007 introduced the hypothesis that an antiangiogenic prolactin fragment with a molecular mass of 16 kDa is a key pathological mediator of peripartum cardiomyopathy (PPCM) (1). The study reported that this fragment is enzymatically generated by the cleavage of full-length prolactin with the lysosomal aspartyl protease cathepsin D. Upon excessive generation, possibly due to high pituitary prolactin secretion near term or *postpartum* and an enhanced oxidative microenvironment, this prolactin fragment would impair myocardial microvascularization and thereby contribute to myocardial dysfunction. Accordingly, a new therapy for PPCM was explored using the dopamine D2 receptor agonists, cabergoline and bromocriptine. Treatment with bromocriptine is currently being evaluated in a multicenter clinical trial (NCT00998556) (2). The concept underlying this putative therapy is the inhibition of the generation of the prolactin fragment by substrate depletion, i.e., the inhibition of pituitary prolactin secretion by activation of dopamine D2 receptors in lactotropes. PPCM is a rare disease which occurred with a frequency of 1 case/3189 live births and an estimated mortality of 1.36–2.05% (confidence interval 0.29–10.8%) from 1990 to 2002 in the United States (3). However, the incidence of PPCM seems to be variable, depending on the geographical region, ethnic background, and other criteria (4, 5).

Since the initial discovery, several research, case report, and review articles have been published (5–10) describing signaling mechanisms mediating the deleterious action of the 16-kDa prolactin fragment and supporting the beneficial effects of treatment with dopamine D2 agonists in patients with PPCM. However, there are relevant aspects to the proposed pathological mechanism in PPCM that are absent in these studies with the consequence of limiting the field by pointing to wrong, or incomplete conclusions.

In contrast to what is suggested in most of the PPCM-related literature, the 16-kDa prolactin fragment is only one of the several antiangiogenic prolactin fragments derived from prolactin *via* cathepsin D and other proteolytic enzymes. Altogether, these fragments of different molecular masses comprise a family of proteins termed vasoinhibins (11, 12). Cathepsin D alone can generate four more vasoinhibins by cleaving full-length prolactin at sites other than the one generating the 16-kDa fragment (13). Three of these cathepsin D-generated vasoinhibin isoforms have documented antiangiogenic activity (11, 13)—a notion that should not go unnoticed when studying the 16-kDa vasoinhibin isoform as a key pathologic mediator of PPCM. The possible contribution of other vasoinhibin isoforms to the pathophysiology of PPCM has neither been investigated nor discussed, not to mention the vasoinhibin isoforms generated by other proteolytic enzymes up regulated in experimental PPCM, such as matrix metalloproteinases (1, 11).

The term “16 kDa PRL” (referring to prolactin as the precursor of the fragment) that has often been used in the PPCM-related literature was updated by the vasoinhibin nomenclature in 2006 (11, 12, 14), and this nomenclature has been refined and reevaluated since then (15–17). The introduction of the vasoinhibin nomenclature was triggered by the recognition that the 16-kDa fragment is not the only endogenous prolactin fragment with antiangiogenic properties. As their functional and structural features are unique and contrast with those of full-length prolactin, it was recognized that these fragments are individual hormones and may not bear the same designation. In consequence they were collectively named “vasoinhibins,” inspired by one of their principal effects, the inhibition of blood vessel growth, and control of blood vessel function.

Another neglected issue concerns the levels of vasoinhibins and the total composition of their isoforms in the circulation during healthy and disease states. In the absence of a quantitative vasoinhibin assay, neither reference ranges nor levels in disease states could be established. Yet, this is required to confirm the contribution of the 16-kDa vasoinhibin isoform to the pathophysiology of PPCM. Higher levels of 16-kDa vasoinhibin would be expected at the onset and declining levels during regression of PPCM. This also concerns the safety of using dopamine agonists to inhibit the generation of vasoinhibins. It should be acknowledged that inhibiting vasoinhibin generation constitutes an intervention into a complex endocrine axis [the prolactin/vasoinhibin axis (18)], which could lead to unintended side effects. Some of the cardiovascular side effects of bromocriptine such as syncope, hypotension, and pleural/pericardial effusion could be influenced by a decline of vasoinhibin levels. This is a possibility as vasoinhibins feature inhibition of vasodilation and vasopermeability (15, 16, 18).

We suggest that whenever the role of the vasoinhibins in PPCM is investigated, their serum levels should be evaluated. This is possible by an established methodology combining immunoprecipitation and western blotting (semi-quantitative) (19). Ideally, at some point in the future, a quantitative vasoinhibin assay could be developed which should then be used to confirm altered levels of vasoinhibin isoforms in PPCM. Although dopamine agonists have been used during pregnancy, the safety of this intervention should carefully be monitored. This is particularly relevant on the background that vasoinhibins are pleiotropic hormones which control angiogenesis-mediated growth in reproductive and non-reproductive organs (18); regulate blood vessel growth, vasopermeability, and vasodilation (15, 16); and have non-vascular effects, which include stimulation of vasopressin release (20), thrombolytic effects (21), and the stimulation of anxiety- and depression-related behaviors (22).

## Author Contributions

JT drafted the manuscript. CC, GE, and TB revised the manuscript.

## Conflict of Interest Statement

The authors declare that the research was conducted in the absence of any commercial or financial relationships that could be construed as a potential conflict of interest.
